# Clinically diagnosed tuberculosis and mortality in high burden settings: a systematic review and meta-analysis

**DOI:** 10.1016/j.eclinm.2025.103251

**Published:** 2025-05-17

**Authors:** Benjamin Freitag, Ayten Sultanli, Maurizio Grilli, Stefan Fabian Weber, Mary Gaeddert, Osman A. Abdullahi, Claudia M. Denkinger, Ankur Gupta-Wright

**Affiliations:** a Division of Infectious Diseases and Tropical Medicine, University of Heidelberg, Germany; b Institute of Tropical Medicine, University of Tübingen, Germany; c Department of Clinical Epidemiology and Applied Biostatistics, Eberhard Karls University, Tübingen, Germany; d German Center for Infection Research (DZIF), Partner Site Tübingen, Tübingen, Germany; e Medical Faculty Mannheim, Library, University of Heidelberg, Germany; f Department for Parasitology, University Hospital Heidelberg, Heidelberg, Germany; g German Center for Infectious Research (DZIF), Partner Site Heidelberg, Heidelberg, Germany; h Department of Public Health, Pwani University, Kilifi, Kenya; i Department of Infectious Diseases, Imperial College London, UK; j Department of Infectious Diseases, North Bristol NHS Trust, Bristol, UK

**Keywords:** Tuberculosis, Clinical diagnosis, Empirical treatment, Diagnosis, Mortality

## Abstract

**Background:**

Clinical diagnosis of tuberculosis (TB), referring to diagnosis without bacteriological confirmation, is common and may affect an individuals’ outcomes. We undertook a systematic review to assess the proportion of people with TB who were diagnosed clinically, and mortality compared to those with bacteriologically confirmation in the published literature.

**Methods:**

We searched Medline, Embase, Web of Science and Cochrane Library from January 2010 to December 2024 using terms for ‘TB’ and diagnostic studies. We excluded studies with participants aged <15 years, not reporting clinical and bacteriologically confirmed TB, not conducted in high TB burden settings, and studies that were not trials, cohort or cross-sectional in design. Published summary data was extracted and risk of bias assessed. Summary estimates for proportion of diagnoses that were clinical were calculated overall and by pre-specified subgroups. Risk ratio for mortality of clinical compared to bacteriological diagnosis was evaluated by random effects meta-analysis. This review was prospectively registered (PROSPERO CRD42023404419).

**Findings:**

Our search identified 5693 records, of which 53 datasets were included. 12 studies were rated as low risk of bias. Median proportion of TB diagnosed clinically (n = 85,623) was 40% (95% CI: 31–46%, interquartile range 27%–53%). The proportion of TB diagnosed clinically was higher in people living with HIV and extrapulmonary TB. Clinical diagnosis did not differ by diagnostic modality available or by study year. The pooled risk ratio for mortality (n = 20,523, 10 studies) was 1·5 (95% CI: 1·0–2·2, *I*^*2*^ = 78·7%) indicating higher mortality in people diagnosed clinically.

**Interpretation:**

Clinical diagnosis of TB remains common and was associated with higher mortality risk than bacteriologically confirmed TB, suggesting conditions other than TB that are not being adequately treated. Better understanding of reasons for clinical TB diagnosis and investment in improved diagnostics for TB and non-TB conditions is needed.

**Funding:**

UK 10.13039/501100000272National Institute for Health and Care Research and 10.13039/501100000691Academy of Medical Sciences; US 10.13039/100000002National Institutes of Health.

**Research in context**

**Evidence before this study**

Current diagnostic tests for tuberculosis (TB) have suboptimal sensitivity, therefore in countries with a high burden of TB the use of ‘clinical diagnosis’, in the absence of bacteriological confirmation, is commonplace. Programmatic data from the World Health Organization estimates approximately 38% of people with TB had bacteriological confirmation in 2023. However, little is known about how this varies in different settings and populations, and with the availability of different diagnostic tests. Furthermore, outcomes for people started on treatment after clinical diagnosis of TB compared to those with bacteriological confirmed TB is not well described. We searched the Medline database for systematic reviews of clinical diagnosis of TB, and found no such studies.

**Added value of this study**

This systematic review included data from 54 studies in high TB burden settings, and found a median of 40% of TB diagnoses. We found higher proportions of clinical diagnosis in people living with HIV, in hospital inpatients, and in extrapulmonary TB. We found mortality was higher in those with clinical TB diagnosis than bacteriologically confirmed TB. Clinical trials of diagnostics interventions showed a reduction in clinical diagnosis with more sensitive diagnostics, but this was not seen in sub-group analysis across observational studies.

**Implications of all the available evidence**

Clinical diagnosis of TB seems to be associated with higher risk of mortality, and is more common in some populations and settings. These data support the need to better understand the reasons for clinical diagnosis of TB, and the need for improved diagnostics.

## Introduction

Despite the global commitment to End Tuberculosis (TB) by 2030, there were still an estimated 1·25 million deaths in 2023 attributed to TB worldwide.^1^ Diagnosis remains the biggest gap in the TB care cascade, and early and accurate diagnosis is key to attain global TB targets of eliminating TB as public health issue.^2^ It is estimated that in 2023 around 2·7 million people worldwide falling sick with TB did not get diagnosed or treated for TB.^1^

The bacteriological detection of TB allows for the correct diagnosis of TB, initiation of the most effective treatment, depending on the presence of drug resistance, and can reduce overtreatment resulting from empirical treatment. For decades, diagnosis of TB relied on sputum smear microscopy, which has suboptimal sensitivity, especially in people living with HIV, extrapulmonary disease and earlier in the disease course.^3^ Therefore, clinical diagnosis (i.e. without a positive bacteriological test for TB) and empirical treatment have been commonplace in practice, and have been important to reduce mortality and morbidity from TB.^4^ Molecular tests for TB are highly specific and more sensitive than sputum microscopy.^5^ Since World Health Organization (WHO) endorsement in 2011, rapid, low complexity molecular tests have been scaled-up by national TB programs, gradually replacing sputum smear microscopy. Despite this, there has not been a substantial increase in the proportion of notified TB that was bacteriologically confirmed since 2010 worldwide-only 62% of pulmonary TB was confirmed bacteriologically in 2023.^1^

Potential reasons for clinical diagnosis of TB include inavailability or delays in bacteriological testing for TB, inability to produce sputum for bacteriological testing, failure to respond to broad-spectrum antibiotics, persistent clinical features compatible with TB despite negative tests, uncertainty about the accuracy of TB tests and a desire to quickly relieve symptoms for patients.^6^, ^7^, ^8^, ^9^ The extensive use of clinical diagnosis for TB has the potential to undermine the impact of more accurate TB diagnostics and could further contribute to overtreatment.^6^^,^^10^^,^^11^ People treated for TB in the absence of bacteriological confirmation may have other conditions missed, and potentially worse outcomes.^12^^,^^13^ However, the drivers of clinical diagnosis, including differential importance between settings and populations are insufficiently understood.

We systematically reviewed and assessed existing evidence for how common clinical diagnosis of TB is, whether this varied by sub-population and setting, and how it affected outcomes compared to outcomes with bacteriologically confirmed TB.

## Methods

For this systematic review and meta-analysis, we describe the proportion of people diagnosed clinically among those diagnosed with TB and, secondarily, the clinical outcomes of those people diagnosed clinically compared to those with a bacteriologically confirmed diagnosis. We defined clinical diagnosis as a diagnosis of TB in the absence of any positive bacteriological test (e.g. sputum smear microscopy, mycobacterial culture, molecular diagnostic test or urinary lipoarbinomannan [LAM] antigen test), either because testing was not done or negative. Clinical diagnosis of TB is synonymous with empirical treatment.^6^^,^^14^

### Search strategy and selection criteria

We systematically searched Medline, Embase, Web of Science and Cochrane Library databases from 1st January 2010 until 31st December 2024 to identify relevant literature. We included studies if they were conducted in 2010 or later, if they were randomized controlled trials (RCTs), cohort studies or cross-sectional studies, written in English, reporting on clinical diagnosis and bacteriological diagnosis, if the included population was older than 15 years and if they were conducted in a WHO high TB burden or WHO high HIV/TB burden country.^15^ 2010 was chosen as the start date as this is when studies of molecular diagnostics in high burden settings were first conducted. Our search strategy combined broad search terms for TB and diagnostic studies (see Appendix pages 2–4 for the details of the search terms). Studies were excluded if they were case control studies, questionnaire based, cost effectiveness studies, enrolled fewer than ten people, community-based or household contact screening or active case finding studies, reporting on a specific or preselected population (e.g. miners, pregnant women, congregate settings), or were studies on tuberculosis meningitis or drug-resistant TB. We did not exclude studies done exclusively in people living with HIV.

Identified records were screened systematically using Rayyan software.^16^ A form was used for title, abstract and full text screening, and studies possibly containing data on clinical diagnosis were selected for full text review. Two authors (BF and AS) conducted screening independently. Conflicts were resolved by consensus, involving third reviewer (AGW) if necessary. After blinded double screening and high agreement for the first 10 studies, 50% of records in title and abstract screen and 50% of records in full-text screen were double screened for quality assurance.

### Data extraction

Data was extracted into a piloted and standardised data extraction form using Covidence software (Veritas Health Innovation, Australia). First and second reviewer extracted data independently, involving third reviewer (AGW) if necessary. To ensure quality and alignment, first and second reviewer double extracted the first 5% of records. Multiple reports on the same study were summarized in a single extraction form where appropriate. Clinical trials with a diagnostic intervention aiming at improving bacteriologically confirmed TB were extracted into different forms for each intervention arm.

The data extracted included study characteristics and design, study population, TB diagnostic tests, and our outcomes of interest (see Appendix page 5). The primary outcome was the proportion of people whose diagnosis of TB was clinical among all people diagnosed with TB. Secondary outcomes were the risk ratio (RR) of mortality between the group of clinically diagnosed compared to bacteriologically diagnosed. Missing Information was not imputed.

### Assessment of bias

We assessed risk of bias using a bespoke tool based on the JBI risk of bias tool for prevalence studies and diagnostic accuracy studies (Appendix Table S1).^17^^,^^18^ Each domain was rated as high risk, low risk or unclear if inadequate information was available. The eight domains were then summarized to a score value (0–8) indicating overall risk for each study. Studies with a score value of 7–8 were rated as low risk studies, studies with a value of 5–6 as high risk staudies and studies with a value ≤4 as very high risk studies. Risk of Bias was assessed independently by first and second reviewer, and conflicts resolved by a third reviewer. Publication bias was assessed by visual inspection of a funnel plot.

### Data analysis and synthesis

Studies meeting our eligibility criteria and reporting data for at least our main objective were used for quantitative data synthesis. Data extracted on absolute numbers of people with clinically diagnosed TB and bacteriologically confirmed TB were used to calculate proportions. Where there was a high degree of heterogeneity between studies on visual assessment of forest plots and using *I*^*2*^, overall summary estimates for the proportion of clinically diagnosed TB are presented as bootstrapped median with 95% confidence interval, supplemented by interquartile ranges.^19^

Heterogeneity was explored by pre-specified subgroup analyses, both within studies (where disaggregated data was available) or at study level. Subgroups included HIV status, pulmonary or extrapulmonary TB, healthcare setting and level (primary i.e. community health centers or outpatients, and secondary i.e. hospitals, inpatients), the main diagnostic methods used (e.g. smear or molecular testing), and if all people were offered bacteriological tests. To investigate the strength of evidence for differences between subgroups, we used random effects meta-analysis, with two-sided alpha at 0·05. To investigate differences over time, we conducted meta-regression of proportion with clinical diagnosis by mean year of study recruitment.

To assess clinical outcomes, disaggregated absolute numbers of people with clinical and bacteriological diagnosis with data on mortality were collected, and RR for mortality calculated. Meta-analysis of binary outcome data was conducted when at least four papers reported on the outcome. We used a random effects model and inverse variance method DerSimonian Laird, combined with Hartung-Knapp adjustment as we expected high degree of heterogeneity.^19^ Meta-regression of the mean year of study recruitment was conducted to further investigate difference over time.

In *post-hoc* analysis, we calculated the absolute proportion reduction in clinical TB diagnosis between trial arms in controlled trials that included a diagnostic intervention aiming at improving bacteriological TB diagnosis. Absolute proportion reduction between the study arms of each clinical trial and standard error were calculated, and data were pooled using a generic inverse variance random effects meta-analysis with Hartung-Knapp adjustment.

Data analysis was conducted using RStudio (version: 2024·04·0 + 73) using *meta*, *metaprop* and *forestploter* packages. Results of individual studies are presented by forest plot. For each meta-analysis conducted, 95% confidence interval as well as prediction interval are reported and *I*^*2*^ statistic used to assess heterogeneity.

We follow the PRISMA 2020 guideline for reporting systematic reviews and meta-analyses.^20^ The protocol for the systematic review was prospectively registered on PROSPERO database (CRD42023404419).

### Role of funding source

No funders had any role in the study design, conduct or analysis.

## Results

**Fig. 1:**
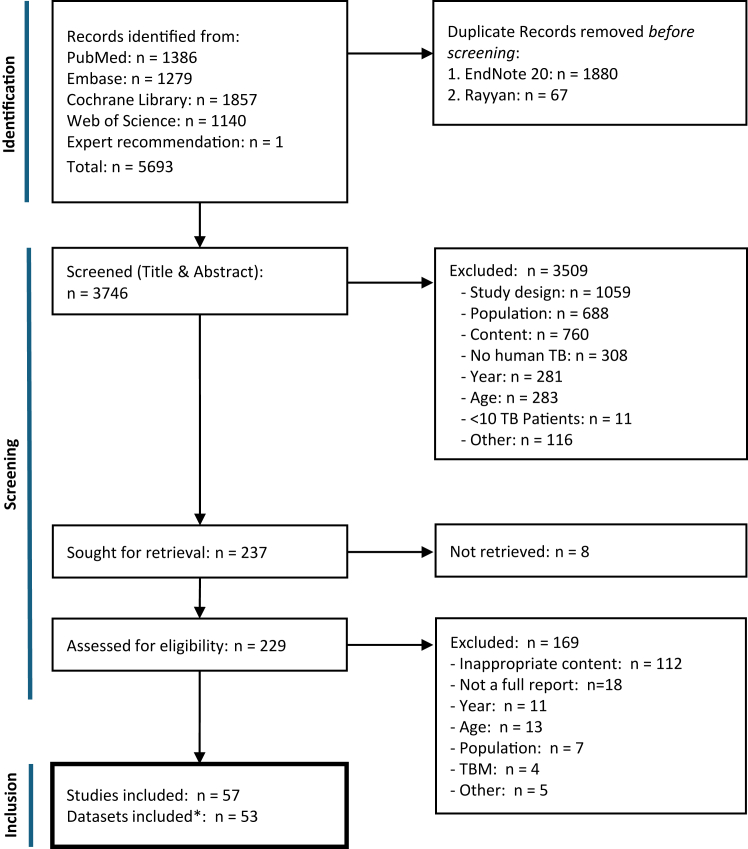
**PRISMA flow diagram**. Flow diagram on study selection process according to PRISMA 2020 guidelines^20^ with list of exclusion reasons. TBM, tuberculosis meningitis, ∗four studies reported the same patient cohort as another included study.

We identified 5693 articles from database searches, which reduced to 3746 after removing duplicates, and to 237 after screening abstract and titles. After full-text review and summarising of multiple articles reporting data on the same cohort, 53 datasets from 57 studies were included in the systematic review (Fig. 1).

The eligible studies included data from 253,829 participants assessed for TB, of whom 85,623 were diagnosed with TB disease and had data on the basis of diagnosis (bacteriologically confirmed or clinically diagnosed). Characteristics are presented in Table 1 and Table S2. The median number of people diagnosed with TB in each study was 420 (range from 38 to 24,265), median age was 37 years (range 30–54 years), and a median of 55% were male (range 31–78%). Most studies (62%) were from the WHO African region, and 17% were from South-East Asia. 53% reported on participants with both extrapulmonary and pulmonary TB, with 30% being limited to pulmonary TB only. The majority of studies were in people with symptoms of TB, although 6 studies included PLHIV irrespective of symptoms. 24% of studies only used sputum smear microscopy as the main diagnostic method for TB, 55% of studies used molecular diagnostics, and 47% included mycobacterial culture. 24% of studies were in a primary healthcare setting, 40% in secondary healthcare. 15% of studies included only inpatients and 24% only outpatients. 38% were cross sectional studies, 34% controlled clinical trials and 23% cohort studies. Overall only 23% of studies had low risk of bias, 77% had high or very high risk of bias (Appendix Table S3).

**Table 1:** Characteristics of studies included in systematic review.

Study ID	RoB/8^a^	Design	Country (-ies)	Healthcare level	Diagnostics available	Population description^b^	Total TB^c^	HIV prevalence^d^
Burke 2024^21^	7	RCT	Malawi	Secondary	CU, XP, LAM, CXR, CAD	≥18 y, PLHIV admitted to hospital, irrespective of TB symptoms, no TB treatment 6 months before enrolment	415	100%
Kibirige 2023^22^	6	DAS	Uganda	Secondary	SM	≥18 y, either TB treatment naïve or initiated on TB treatment	232	38·6%
Sachdeva 2015^23^	6	CHS	India	Primary	SM, XP, CXR	TB symptoms and producing sputum	17,587	–
Adewole 2015^24^	5	RCT	Nigeria	Secondary	SM	≥15 y, not on TB treatment, no relevant coexisting medical conditions	150	6·7%
Balcha 2014^25^	6	CHS	Ethiopia	Primary	CU >50%, SM, XP	Adult PLHIV, not on ART, irrespective of TB symptoms, producing sputum	158	100%
Durovni 2014^26^	4	CRT	Brazil	Mixed	SM, CU, XP, CXR	Producing sputum, PTB only	4660	8·9%
McCarthy 2018^27^^,^^28^	5	CRT	South Africa	Primary	SM, CU, XP, CXR	≥18 y, started on TB treatment, negative initial XTEND study sputum results, clinical indication for sputum investigation	541	62%
Bekele 2018^29^	4	RCT	Ethiopia	Mixed	CU >50%, SM, IGRA	Adults, newly diagnosed PTB, no relevant coexisting medical conditions	348	0%
Eneogu 2024^30^	3	CSS	Nigeria	–	–	People diagnosed with TB, data from laboratory register	4823	–
Cox 2014^31^	7	RCT	South Africa	Primary	SM, CU, XP	Adults, TB symptoms, no TB Treatment for >3 d before enrolment	506	58·8%
Majella 2021^32^	6	RCT	India	Secondary	SM	New diagnosis of TB, possession of mobile phone	310	5·8%
DeCastro 2021^33^	4	RCT	Brazil, Côte d’Ivoire, +3	–	SM, CU, XP, LAM, CXR	≥18 y, ART naive, Rifampicin containing TB Treatment initiated within 2 months, no relevant coexisting medical conditions	457	100%
Rima 2024^34^	7	CSS	Ethiopia	Mixed	SM, XP, CXR	≥15 y, PTB, on directly observed therapy, mainly rural, not critically ill	393	–
Dave 2013^35^	7	CSS	India	Mixed	SM	Adults and children with TB diagnosis	556	4·3%
Gupta-Wright 2018^36^	7	RCT	Malawi, South Africa	Secondary	SM, CU, XP, LAM, CXR	≥18 y, hospitalized, urban and rural, irrespective of TB symptoms, not on TB treatment for 12 months before enrolment	474	100%
Hanifa 2016^37^	4	CHS	South Africa	–	SM, CU, XP, LAM, CXR	≥18 y, CD4 count <200 cells/μl, in HIV care, irrespective of TB symptoms, no TB treatment within 3 months before enrolment	56	100%
Getahun 2016^38^	6	CSS	Ethiopia	–	SM	Adults, new TB cases, on directly observed therapy at least for 1 month	576	–
Atekem 2018^39^	6	CSS	Cameroon	Primary	SM	–	895	47·7%
Ereso 2024^40^	4	CSS	Ethiopia	Mixed	–	≥15 y, initiated TB treatment	755	3·3%
Weber 2018^41^	6	CHS	India	Secondary	SM, CU, CXR	≥16 y, TB symptoms	285	14%
Cattamanchi 2021^42^	6	RCT	Uganda	Primary	SM, XP	Adults, PTB, [we included people started on treatment only]	824	43·8%
Prudhivi 2019^43^	7	CHS	India	Secondary	SM	New and retreatment PTB, data on treatment outcome available	1113	23%
Åhsberg 2023^44^	7	CRT	Ghana	Secondary	CU, XP, LAM	Adult PLHIV, TB symptoms, severe illness or advanced HIV, not receiving TB treatment in the preceding 60 days	105	100%
Padda 2015^45^	6	CSS	India	–	SM	TB treatment directly observed therapy started	2571	–
Manosuthi 2012^46^	5	RCT	Thailand	Secondary	SM, CU, CXR	18–65 y, CD4 count <350 cells/μl, ART naive, no relevant coexisting medical conditions	156	100%
Bock 2018^47^	5	CHS	South Africa	Primary	CU, XP, CXR	≥18 y, started ART	97	100%
Theron 2014^48^^,^^49^	8	RCT	South Africa, Zimbabwe, +2	Primary	CU >50%, SM, XP, CXR	≥18 y, periurban, TB symptoms, no treatment in previous 60 d, producing sputum	645	59·6%
Bjerrum 2015^50^	6	CSS	Ghana	Secondary	SM, CU, XP, CXR, LAM	≥18 y, CD4 count ≤350 cells/μl, irrespective of TB symptoms, producing sputum, no TB treatment 3months before enrolment	100	100%
Shivalingaiah 2024^51^	5	CHS	India	–	–	All TB cases ≥18 y, registered for treatment at study site	516	1·7%
Hanrahan 2013^52^	7	CHS	South Africa	Primary	CU >50%, SM, XP	TB symptoms, informal settlement communities	116	69·1%
Zerihun 2023^53^	7	CSS	Ethiopia	Primary	SM	≥18 y, PTB, newly started on TB treatment, mainly urban	636	16·7%
Shrivastava 2013^54^	6	CHS	India	Secondary	SM	TB symptoms	61	10·2%
Bezerra 2020^55^	4	CHS	Brazil	Secondary	–	Adults, started on TB Treatment	148	37·2%
Songkhla 2019^56^	6	CHS	Thailand	Secondary	CU >50%, SM, LAM, CXR	Adults, CD4 cell count ≤200/μl, symptomatic, irrespective of sputum production, no TB Treatment within 3 months before enrolment	137	100%
Jiang 2023^57^	5	CSS	China	–	SM, CU, XP	Not on TB treatment yet, not recorded as “dead”, “treatment failure”, or who were “not evaluated”	24,265	–
Hemalatha 2023^58^	3	CSS	India	Secondary	–	PTB and EPTB	45	–
Abdullahi 2021^12^	7	CSS	Kenya	Mixed	SM, XP, CXR	Adults, started on TB treatment	12,856	29%
Kaku 2024^59^	4	CSS	Indonesia	–	XP	–	158	1·2%
Jin 2020^60^	5	DAS	China	Secondary	CU >50%, XP	People with different diseases (infectious and non-infectious)	125	–
Ncube 2019^61^	5	CSS	Zimbabwe	Primary	–	≥15 y, newly registered, densely populated poor urban suburb resident	1617	67·5%
Peter 2016^62^^,^^63^	6	RCT	South Africa, Tanzania, +2	Secondary	CU >50%, SM, XP, LAM, CXR	≥18 y, symptomatic, severely ill, no TB treatment within 60 d before testing	1246	100%
Gebreegziabher 2016^64^	6	CSS	Ethiopia	Mixed	SM	≥15 y, newly diagnosed PTB, no TB retreatment cases	706	11·6%
Mupfumi 2014^65^	6	RCT	Zimbabwe	Secondary	SM, XP, CXR	≥18 y, ART naive initiating ART, not on TB treatment, urban resident, irresp. of TB symptoms	88	100%
Mohammed 2020^66^	4	CSS	Ethiopia	Mixed	SM, XP, CXR	TB symptoms	2483	12·5%
Qiu 2015^67^	6	RCT	China	Secondary	CU >50%, SM, CXR, IGRA	TB symptoms	597	0%
Seid 2018^68^	5	CSS	Ethiopia	Mixed	SM, XP, CXR	≥18 y, newly diagnosed TB, <15 d of TB treatment, not critically ill	382	20·4%
Kebede 2021^69^	7	CSS	Ethiopia	Secondary	SM	≥15 y, started on TB treatment	465	27·4%
Getiye 2024^70^	6	CSS	Ethiopia	Mixed	–	Adults, newly diagnosed PTB attending TB clinics in public health facilities, mainly rural	420	2%
Auld 2016^71^	7	CHS	Cambodia	Mixed	SM, CU, XP, CXR	PLHIV, rural, symptomatic	234	100%
O'Connor 2017^72^	6	CRT	Lesotho	Mixed	SM, CU, XP, CXR	≥18 y, newly registered for TB treatment	1233	100%
Mishra 2023^73^	5	CSS	India	Secondary	SM	Adult, on DOTS, new cases of PTB, on treatment for <10 days, no relevant coexisting medical conditions	341	0%
Humphrey 2020^74^	5	CHS	Kenya, Uganda, +10	Secondary	SM, CU, XP, LAM, CXR	≥15 y, started on TB treatment	2091	100%
Beckwith 2021^75^^,^^76^	6	RCT	South Africa	Secondary	SM, CU, XP, CXR	≥18 y, CD4 count ≤150 cells/μl, no relevant coexisting medical conditions, newly diagnosed TB	95	100%

CSS, cross sectional; RCT, randomised controlled trial; DAS, diagnostic accuracy study; CRT, cluster randomized trial; SM, smear microscopy; CU, culture; XP, X-pert; CXR, chest X-ray; IGRA, interferon gamma release assay; LAM, lipoarabinomannan on urine; CAD, digital chest X-ray with computer-aided diagnosis; PLHIV, people living with HIV; PHC, primary health care; ART, antiretroviral therapy; DOTS, direct observed therapy.

a Risk of Bias Score out of 8, higher scores indicate lowest risk of bias.

b Population description as described in the study manuscript. TB symptoms refer to cough, fever, weight loss or night sweats.

c Total amount of people diagnosed with TB.

d HIV prevalence in the study population.

**Fig. 2:**
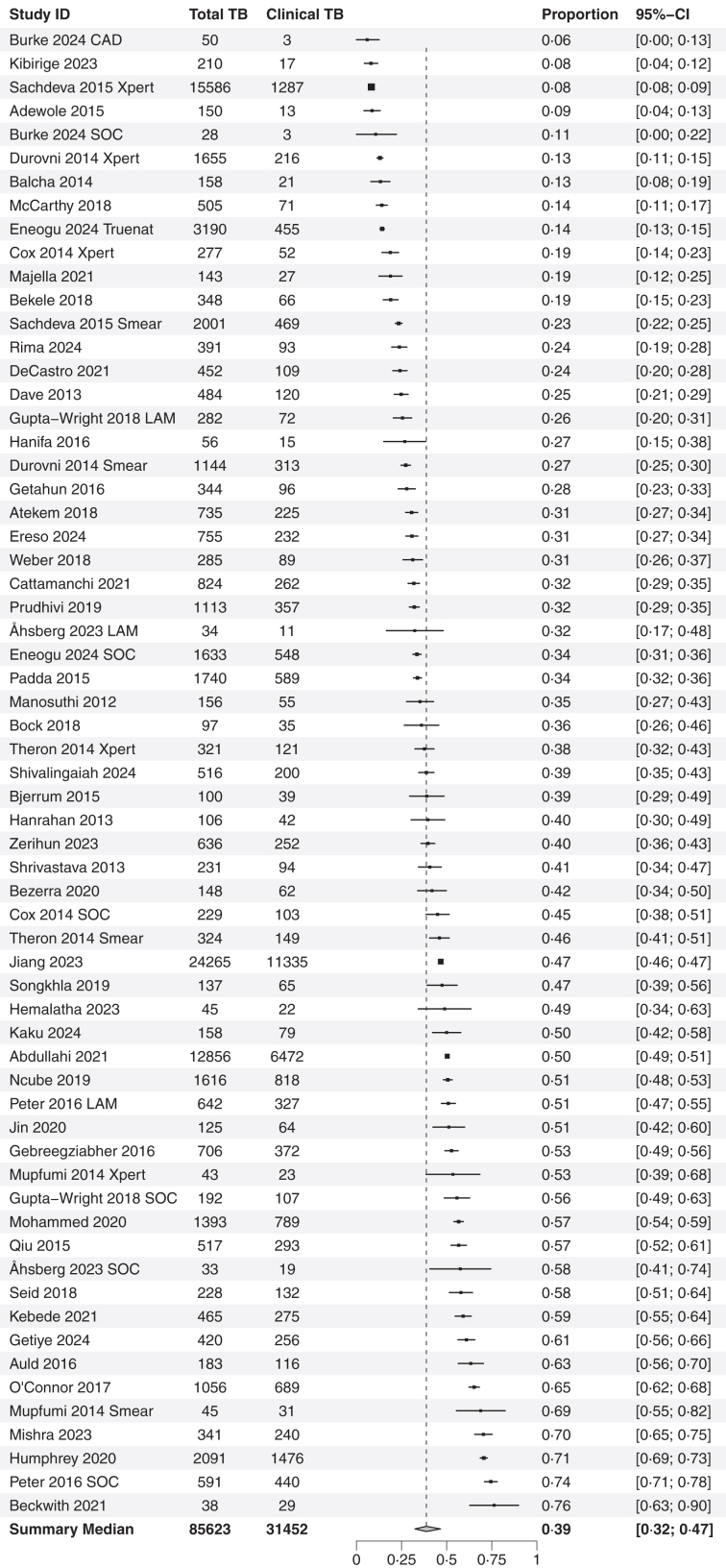
**Proportion of clinical TB**. Forest Plot on the proportion of clinically diagnosed TB people in all people diagnosed as TB with data on confirmation status available. Overall proportion with 95% CI below is the bootstrapped median of data above. Absolute numbers of people diagnosed with TB (Total TB) and clinically diagnosed TB (Clinical TB) are on the left, and proportion and 95% CI on the right. Where clinical trial arms have been presented separately, the description of the arm is presented after the citation: SOC Standard of Care, Smear is sputum smear microscopy, Xpert is Xpert MTB/RIF or Xpert MTB/RIF Ultra arm, LAM is Urine lipoarabinaomannan (LAM) testing, CAD is digital chest X-ray with computer-aided diagnosis and Truenat is Truenat real time PCR device.

The median overall proportion of study participants with a clinical diagnosis of TB was 40% (95% CI: 31–46%, IQR: 27%–53%, n = 63, Fig. 2), with high levels of heterogeneity between studies (*I*^*2*^ = 99%, Figure S1). The proportion of clinical diagnosis was substantially higher for extrapulmonary TB (78%, 95% CI: 48–93%, n = 6, Figure S2), compared to only pulmonary TB (34%, 95% CI: 28–40%, n = 35, p = 0·001). Similarly, clinical diagnosis was more common in people living with HIV (45%, 95% CI: 38–52%, n = 35, Figure S4), compared to those not living with HIV (33%, 95% CI: 25–42%, n = 15, p = 0·026 Figure S5). TB was also diagnosed clinically more often in secondary care compared to primary care (47% versus 34%, p = 0·01, Figure S6), and in inpatients compared to outpatients (51% versus 40%, p = 0·29, Figure S7).

Clinical diagnosis was more common in studies where not all participants received bacteriological testing compared to studies where all participants had at least one bacteriological test (60% versus 35%, p < 0·001, Figure S8). The proportion of clinical TB did not differ by whether studies used sputum smear microscopy or molecular tests as the main diagnostic test for TB (Figure S9), or by risk of bias score (Figure S10). Meta-regression by year of study found no changes over time (Figure S11), and there was no evidence of publication bias (Figure S12).

**Fig. 3:**
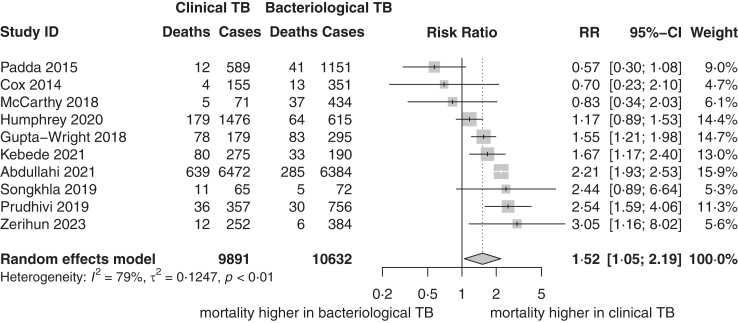
**Mortality of clinical TB**. Forest plot depicting random effects inverse variance meta-analysis on mortality risk ratio of clinically diagnosed TB people compared to bacteriologically confirmed TB people with data on confirmation status available. Absolute numbers on the left are deaths and total number of people (cases) with clinical or bacteriological diagnosis. RR per study with 95% CIs and weight on the right. Pooled RR with 95% CI displayed below as well as heterogeneity assessment. Median duration of follow-up is 6·0 months (IQR 4·5 months).

Mortality data stratified by clinical or bacteriologically confirmed TB was available for ten studies including 20,523 participants, of whom 48% were diagnosed clinically and 52% bacteriologically. The RR for mortality varied from 0·6 to 3·1, with three studies reporting lower mortality risk in people with clinical TB diagnosis. The pooled RR was 1·5 (95% CI: 1·1–2·2, *I*^*2*^ = 78, 7%, Fig. 3), indicating a higher mortality risk in those with a clinical diagnosis. For studies with a low risk of bias, pooled RR was higher than in studies with high risk of bias (RR 1·9, 95% CI: 1·4–2·7, n = 6 compared to 1·0, 95% CI: 0·4–2·4, n = 4 respectively, p = 0·04, Figure S13). Meta-regression by year found a positive correlation, with a higher risk ratio for mortality in more recent years (p = 0·004, Figure S14), with more recent studies having a two-fold higher RR.

**Fig. 4:**
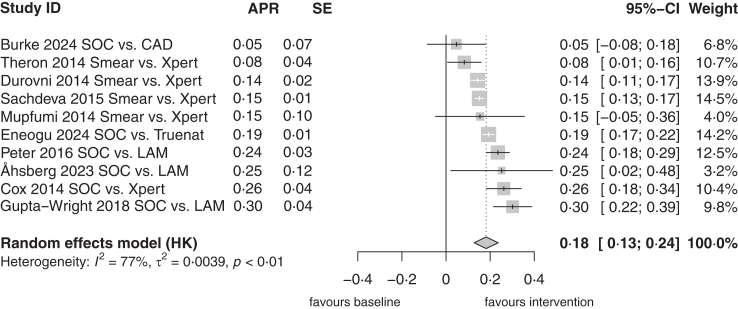
**Reduction of porportion of clinical TB in clinical trials with diagnostic intervention**. Forest plot depicting inverse variance random effects meta-analysis on absolute proportion reduction (APR) between the two arms of clinical trials aiming on the reduction of the proportion of clinical TB in their diagnostic intervention. Pooled APR indicates, that interventions of clinical trials reduced the proportion of clinical TB. The APR and standard error (SE) on the left. APR with 95% CI and weight on the right. Below the pooled estimate with 95% CI as well as heterogeneity assessment. SOC is Standard of Care, Smear is sputum smear microscopy, Xpert is Xpert MTB/RIF or Xpert MTB/RIF Ultra arm, and LAM is Urine lipoarabinaomannan (LAM) testing. CAD is digital chest X-ray with computer-aided diagnosis and Truenat is Truenat real time PCR device.

We identified ten clinical trials of diagnostic interventions aiming to improve bacteriological diagnosis, including trials of implementation of Xpert MTB/RIF and/or urine lipoarabinomannan assays (Appendix Table S4). All studies demonstrated a decrease in proportion of clinically diagnosed TB in the intervention arms compared to standard of care, which varied from 5% to 30%, with a pooled absolute reduction of 18% (95% CI: 13–24%, Fig. 4).

## Discussion

We report, to our knowledge, the first systematic review to assess clinical diagnosis of TB and associated mortality in high burden settings. Our main findings are that clinical diagnosis of TB remains common, occurring in a median of 40% of people diagnosed with TB, and has not decreased over time or by availability of molecular diagnostic tests. There was high variability between studies, but clinical diagnosis was more common in people living with HIV, in secondary care and inpatients, and in extrapulmonary TB. Mortality risk was approximately 50% higher in those with clinical diagnosis compared to those with bacteriological confirmation.

Our findings concur with programmatic data reported in the WHO Global TB report, with the most recent global data reporting only 62% of pulmonary TB was bacteriologically confirmed in 2023.^1^ The implementation of more sensitive molecular diagnostics should, theoretically, lead to a decrease in the need for clinical diagnosis and empirical treatment compared to only having sputum smear microscopy available. However, based on both our results, and programmatic data collated by WHO, this is yet to be seen. While the impact might present only slowly over time, we did not observe a trend in our data. In contrast, we found clinical trials comparing more sensitive TB diagnostic tests (predominantly Xpert MTB/RIF and urine lipoarabinomannan antigen tests) to standard demonstrated a median absolute reduction in clinical TB diagnosis of 19%. This discrepancy between clinical trials, and non-interventional study and programmatic data might partially be explained by a study (Hawthorne) effect,^77^ where healthcare workers in trials are aware that decisions about TB diagnosis are being observed.

Whilst the introduction of more sensitive tests should decrease the proportion of people treated for TB based on clinical diagnosis, public health interventions to reduce TB incidence may have the opposite effect. Data from Blantyre, Malawi, found clinically diagnosed TB did not fall to the same degree as bacteriologically confirmed TB in the context of reducing TB incidence between 2011 and 2019, suggesting significant overtreatment.^78^ The decision to start TB treatment in the absence of a positive bacteriological test is complex, and will be affected by poorly quantifiable factors including national and local guidelines and epidemiology, access to other diagnostics (e.g. radiology, microbiology), and individual health worker factors (e.g. training and clinical experience). Clinicians may have a high index of suspicion for diagnosing TB and thus a low threshold for starting TB treatment. Improving healthcare workers’ knowledge on pretest probability of TB, including the impact of more sensitive diagnostics and changing incidence, may reduce overtreatment through clinical diagnosis.^79^ However, if people are diagnosed earlier in the disease course with more paucibacillary disease, this may also reduce the sensitivity of diagnostic tests for TB.

We found higher proportions of clinical diagnosis amongst people living with HIV and extrapulmonary TB. Sputum based diagnostics are known to be less sensitive in people living with HIV, although the difference in sensitivity is less for newer molecular assays such as Xpert MTB/RIF Ultra (Cepheid, USA) compared with sputum smear microscopy.^80^ This, coupled with high mortality associated with missed TB in people living with HIV, is likely driving more clinical diagnoses and empirical treatment.^4^^,^^13^ In studies not able to test all those with presumptive TB using bacteriological tests, the proportion of clinical diagnoses was also higher than in studies testing everyone. This may be due to challenges in obtaining sputum samples for TB testing.^81^ However, even when everyone underwent bacteriological testing, one-third of diagnoses were clinical. Together, these findings strongly support the need for TB diagnostics that (1) do not solely rely on sputum, (2) are easily implementable at peripheral levels of healthcare, and (3) can accurately diagnose extrapulmonary and HIV-associated TB.^82^

Our results found people diagnosed clinically with TB had worse outcomes, including a 50% higher relative risk of mortality. One possible explanation is other undiagnosed conditions that are not treated, leading to morbidity and mortality.^83^, ^84^, ^85^, ^86^ This was even more marked in people living with HIV, where other opportunistic infection can mimic TB clinically. As the incidence of TB decreases, there is a need for improved access to non-TB diagnostics for people presenting to healthcare facilities in high TB burden settings with presumptive TB, especially at peripheral healthcare levels. This could help reduce morbidity and mortality associated with clinic diagnosis of TB. Alternative mechanisms for the association with mortality is that more critically unwell individuals are more likely to have difficulty in producting sputum for testing, and therefore are more likely to be treated for TB based on clinical diagnosis. It is not clear why mortality risk was higher in more recent studies, it maybe that as diagnostics have improved a lower proportion of those with clinically diagnosed TB actually have TB.

Strengths of this systematic review include the large number of studies identified for the main analysis, representing a wide range of settings, and allowing subgroup analyses and meta regression. A further strength is our analysis of mortality outcomes. Our main limitation is the high degree of heterogeneity, likely resulting from differing practice, availability of TB diagnostics (e.g. smear versus molecular diagnsotics, the use of urinary LAM antigen tests) procedures and TB prevalence across settings and studies. The reasons for clinical diagnosis, tests performed as part of diagnostic algorithm and criteria for initiation of TB treatment could not be investigated in more detail. We did not undertake subgroup analysis by region as the majority of studies were from the African region. The nature of many studies indicated high risk of bias, although our findings did not change significantly when limiting to low risk of bias studies (Appendix Figure S10). We were unable to report any outcomes other than mortality due to data availability, and follow-up time varied between studies.

In conclusion, we found clinical diagnosis of TB to be commonplace despite implementation of more sensitive molecular diagnostics. Clinical diagnosis was also associated with higher mortality. There is a need to better understand the reasons for clinical TB treatment in high burden settings, and investment in improved diagnostics for TB and non-TB conditions.

## Contributors

AGW and CD developed the idea and initiated the article. BF and AGW drafted study design and protocol with input from AS, MGaeddart and CD. The search was designed and conducted by BF, MGrilli and AGW. Screening, data extraction and risk of bias assessment was done by BF, AS and AGW. BF and AGW undertook the data analysis. BF, AGW, MGaeddart, OA, SW and CD interpreted results, BF and AGW wrote the first draft of the manuscript. All authors contributed to the manuscript by edits and providing critical feedback. All authors had full access to the data and had final responsibility for submission.

## Data sharing statement

This manuscript is based on secondary data which is published and publicaly available. Data extracted for the analysis is presented within the results section and Supplementary Appendix.

All data reported in this study derives from published studies cited in the reference section. Our data set will not be made available public. The code used for the analyses is available upon request.

## Declaration of interests

There are no conflicts of interest to declare.
