# Supplementary material for: Clinically diagnosed tuberculosis and mortality in high burden settings: a systematic review and meta-analysis
Source: eClinicalMedicine. 2025 May 17;84:103251. doi: 10.1016/j.eclinm.2025.103251 (PMC12146525; doi:10.1016/j.eclinm.2025.103251)
Supplement: Appendix [file mmc1.docx]

**Supplementary Appendix**

Clinically diagnosed tuberculosis and mortality in high burden settings: a systematic review and meta-analysis.

**Index:**

[**Search Strategy** 2](#_Toc184047858)

[**Table S1: tool for risk of bias evaluation.** 5](#_Toc184047859)

[**List of variables extracted** 5](#_Toc184047860)

[**Table S2: Extended baseline table of included studies** 9](#_Toc184047861)

[**Table S3: Risk of bias assessment results** 10](#_Toc184047862)

[**Figure S1: Meta-analysis of proportion of clinical TB diagnosis for all studies** 11](#_Toc184047863)

[**Figure S2: Meta-analysis of proportion of clinical TB diagnosis in extrapulmonary TB** 12](#_Toc184047864)

[**Figure S3: Meta-analysis of proportion of clinical TB diagnosis in pulmonary TB** 13](#_Toc184047865)

[**Figure S4: Meta-analysis of proportion of clinical TB diagnosis in people living with HIV** 14](#_Toc184047866)

[**Figure S5: Meta-analysis of proportion of clinical TB diagnosis in people not living with HIV** 15](#_Toc184047867)

[**Figure S6: Meta-analysis of proportion of clinical TB diagnosis by healthcare level** 16](#_Toc184047868)

[**Figure S7: Meta-analysis of proportion of clinical TB diagnosis in subgroups by clinical setting** 17](#_Toc184047869)

[**Figure S8: Meta-analysis of proportion of clinical TB diagnosis by availability of bacteriologial testing** 18](#_Toc184047870)

[**Figure S9: Meta-analysis of proportion of clinical TB diagnosis by diagnostic method available** 19](#_Toc184047871)

[**Figure S10: Meta-analysis of proportion of clinical TB diagnosis by risk of bias assessment** 20](#_Toc184047872)

[**Figure S11: Meta-regression of proportion of clinical TB diagnosis over mean year of study period** 21](#_Toc184047873)

[**Figure S12: Funnel Plot on identified studies** 22](#_Toc184047874)

[**Figure S13: Meta-analysis of mortality risk by risk of bias assessment** 23](#_Toc184047875)

[**Figure S14: Meta-regression of mortality risk over time** 24](#_Toc184047876)

[**Table S4: Diagnostics used in study arms in clinical trials with an intervention aiming at the reduction of the proportion of clinical TB** 25](#_Toc184047877)

###

**Supplementary Methods**

### **Search Strategy**

**PubMed**

**P**

| 1 | **"Tuberculosis"[Mesh] OR** Tuberculo*[tw] OR TB[tw] |
| --- | --- |

**I**

| 2 | **"Diagnosis"[Mesh] OR** Diagnos*[tw] OR Test*[tw] OR Culture*[tw] OR Smear*[tw] OR Xpert*[tw] |
| --- | --- |

**C**

| 3 | "Angola"[Mesh] OR "Bangladesh"[Mesh] OR "Botswana"[Mesh] OR "Brazil"[Mesh] OR "Cambodia"[Mesh] OR "Cameroon"[Mesh] OR "Central African Republic"[Mesh] OR "Chad"[Mesh] OR "China"[Mesh] OR "Congo"[Mesh] OR "Democratic Republic of the Congo"[Mesh] OR "Democratic People's Republic of Korea"[Mesh] OR "Eswatini"[Mesh] OR "Ethiopia"[Mesh] OR "Gabon"[Mesh] OR "Guinea"[Mesh] OR "Guinea-Bissau"[Mesh] OR "Ghana"[Mesh] OR "India"[Mesh] OR "Indonesia"[Mesh] OR "Kenya"[Mesh] OR "Lesotho"[Mesh] OR "Liberia"[Mesh] OR "Malawi" [Mesh] OR "Mongolia"[Mesh] OR "Mozambique"[Mesh] OR "Myanmar"[Mesh] OR "Namibia"[Mesh] OR "Nigeria"[Mesh] OR "Pakistan"[Mesh] OR "Papua New Guinea"[Mesh] OR "Philippines"[Mesh] OR "Russia"[Mesh] OR "Sierra Leone"[Mesh] OR "South Africa"[Mesh] OR "Tanzania"[Mesh] OR "Thailand"[Mesh] OR "Uganda"[Mesh] OR "Vietnam"[Mesh] OR "Zambia"[Mesh] OR "Zimbabwe"[Mesh] OR angola*[tw] OR bangladesh*[tw] OR botswan*[tw] OR batswan*[tw] OR motswan*[tw] OR brazil*[tw] OR cambodia*[tw] OR cameroon*[tw] OR "central africa*"[tw] OR chad*[tw] OR chin*[tw] OR congo*[tw] OR kinshasa*[tw] OR brazzaville*[tw] OR "north korea*"[tw] OR eswatin*[tw] OR emaswati*[tw] OR swaziland*[tw] OR ethiopia*[tw] OR gabon*[tw] OR ghana*[tw] OR guinea*[tw] OR india*[tw] OR indonesia*[tw] OR kenya*[tw] OR lesotho*[tw] OR sotho[tw] OR liberia*[tw] OR malawi*[tw] OR mongolia*[tw] OR mozambi*[tw] OR myanmar*[tw] OR burm*[tw] OR namibia*[tw] OR nigeria*[tw] OR pakistan*[tw] OR "papua new guinea*"[tw] OR philippine*[tw] OR filipino[tw] OR russia*[tw] OR "sierra leon*"[tw] OR "south africa*"[tw] OR tanzania*[tw] OR thai*[tw] OR uganda*[tw] OR vietnam*[tw] OR zambia*[tw] OR zimbabwe*[tw] OR Africa*[tw] OR Asia*[tw] |
| --- | --- |

**Clinical Trials**

Box 3.b Cochrane Highly Sensitive Search Strategy for identifying randomized trials in MEDLINE: sensitivity- and precision-maximizing version; PubMed format, S. 60-61

**https://training.cochrane.org/technical-supplement-chapter-4-searching-and-selecting-studies-v63**

| 4 | clinical trials as topic [mesh:noexp] OR randomized controlled trial[pt] OR controlled clinical trial[pt] OR randomized[tiab] OR placebo[tiab] OR randomly[tiab] OR trial[ti] |
| --- | --- |

**Strings**

1-4 as in the tables above

| 5 | 1 AND 2 AND 3 AND 4 |
| --- | --- |

**Publications from 2010**

| 6 | 5 AND 2010/1/1:3000/12/12[pdat] |
| --- | --- |

**Embase**

**P**

| 1 | 'tuberculosis'/exp OR Tuberculo*:ti,ab,kw OR TB:ti,ab,kw |
| --- | --- |

**I**

| 2 | 'diagnosis'/exp OR Diagnos*:ti,ab,kw OR Test*:ti,ab,kw OR Culture*:ti,ab,kw OR Smear*:ti,ab,kw OR Xpert*:ti,ab,kw |
| --- | --- |

**C**

| 3 | 'Angola'/exp OR 'Bangladesh'/exp OR 'Botswana'/exp OR 'Brazil'/exp OR 'Cambodia'/exp OR 'Cameroon'/exp OR 'Central African Republic'/exp OR 'Democratic Republic Congo'/exp OR 'North Korea'/exp OR 'Eswatini'/exp OR 'Ethiopia'/exp OR 'Gabon'/exp OR 'Guinea'/exp OR 'Guinea-Bissau'/exp OR 'Ghana'/exp OR 'India'/exp OR 'Indonesia'/exp OR 'Kenya'/exp OR 'Lesotho'/exp OR 'Liberia'/exp OR 'Malawi'/exp OR 'Mongolia'/exp OR 'Mozambique'/exp OR 'Myanmar'/exp OR 'Namibia'/exp OR 'Nigeria'/exp OR  'Pakistan'/exp OR 'Papua New Guinea'/exp OR 'Philippines'/exp OR 'Russian Federation'/exp OR 'Sierra Leone'/exp OR 'South Africa'/exp OR 'Tanzania'/exp OR 'Thailand'/exp OR 'Uganda'/exp OR 'Viet Nam'/exp OR 'Zambia'/exp OR 'Zimbabwe'/exp OR angola*:ti,ab,kw OR bangladesh*:ti,ab,kw OR botswan*:ti,ab,kw OR batswan*:ti,ab,kw OR motswan*:ti,ab,kw OR brazil*:ti,ab,kw OR cambodia*:ti,ab,kw OR cameroon*:ti,ab,kw OR "central africa*":ti,ab,kw OR chad*:ti,ab,kw OR chin*:ti,ab,kw OR congo*:ti,ab,kw OR kinshasa*:ti,ab,kw OR brazzaville*:ti,ab,kw OR "north korea*":ti,ab,kw OR eswatin*:ti,ab,kw OR emaswati*:ti,ab,kw OR swaziland*:ti,ab,kw OR ethiopia*:ti,ab,kw OR gabon*:ti,ab,kw OR ghana*:ti,ab,kw OR guinea*:ti,ab,kw OR  india*:ti,ab,kw OR indonesia*:ti,ab,kw OR kenya*:ti,ab,kw OR lesotho*:ti,ab,kw OR sotho:ti,ab,kw OR liberia*:ti,ab,kw OR malawi*:ti,ab,kw OR mongolia*:ti,ab,kw OR mozambi*:ti,ab,kw OR myanmar*:ti,ab,kw OR burm*:ti,ab,kw OR namibia*:ti,ab,kw OR nigeria*:ti,ab,kw OR pakistan*:ti,ab,kw OR "papua new guinea*":ti,ab,kw OR philippine*:ti,ab,kw OR filipino:ti,ab,kw OR russia*:ti,ab,kw OR "sierra leon*":ti,ab,kw OR "south africa*":ti,ab,kw OR tanzania*:ti,ab,kw OR thai*:ti,ab,kw OR uganda*:ti,ab,kw OR vietnam*:ti,ab,kw OR zambia*:ti,ab,kw OR zimbabwe*:ti,ab,kw OR Africa*:ti,ab,kw OR Asia*:ti,ab,kw |
| --- | --- |

**Clinical Trials**

Box 3.e Cochrane Highly Sensitive Search Strategy for identifying controlled trials in Embase: (2020 revision); Embase.com format. S. 63-64

<https://training.cochrane.org/technical-supplement-chapter-4-searching-and-selecting-studies-v63>

| 4 | (‘randomized controlled trial’/de OR ‘controlled clinical trial’/de OR random*:ti,ab,tt OR ‘randomization’/de OR ‘intermethod comparison’/de OR placebo:ti,ab,tt OR (compare:ti,tt OR compared:ti,tt OR comparison:ti,tt) OR ((evaluated:ab OR evaluate:ab OR evaluating:ab OR assessed:ab OR assess:ab) AND (compare:ab OR compared:ab OR comparing:ab OR comparison:ab)) OR (open NEXT/1 label):ti,ab,tt OR ((double OR single OR doubly OR singly) NEXT/1 (blind OR blinded OR blindly)):ti,ab,tt OR ‘double blind procedure’/de OR (parallel NEXT/1 group*):ti,ab,tt OR (crossover:ti,ab,tt OR ‘cross over’:ti,ab,tt) OR ((assign* OR match OR matched OR allocation) NEAR/6 (alternate OR group OR groups OR intervention OR interventions OR patient OR patients OR subject OR subjects OR participant OR participants)):ti,ab,tt OR (assigned:ti,ab,tt OR allocated:ti,ab,tt) OR (controlled NEAR/8 (study OR design OR trial)):ti,ab,tt OR (volunteer:ti,ab,tt OR volunteers:ti,ab,tt) OR ‘human experiment’/de OR trial:ti,tt) NOT (‘animal experiment’/de NOT (‘human experiment’/de OR ‘human’/de)) |
| --- | --- |

**Strings**

1-4 as in the tables above

| 5 | 1 AND 2 AND 3 AND 4 |
| --- | --- |

**To switch off PubMed**

| 6 | 5 NOT ([medline]/lim OR [pubmed-not-medline]/lim) |
| --- | --- |

**To exclude document types not indexed in PubMed**

| 7 | 6 NOT ('Conference Abstract'/it OR 'Note'/it OR 'chapter'/it) |
| --- | --- |

**Publications from 2010**

| 8 | publication date from Jan 2010 to present |
| --- | --- |

**Cochrane Library**

**P**

| 1 | **[mh "Tuberculosis"] OR** Tuberculo*:ti,ab,kw OR TB:ti,ab,kw |
| --- | --- |

**I**

| 2 | **[mh "Diagnosis"] OR** Diagnos*:ti,ab,kw OR Test*:ti,ab,kw OR Culture*:ti,ab,kw OR Smear*:ti,ab,kw OR Xpert*:ti,ab,kw |
| --- | --- |

**C**

| 3 | [mh "Angola"] OR [mh "Bangladesh"] OR [mh "Botswana"] OR [mh "Brazil"] OR [mh "Cambodia"] OR [mh "Cameroon"] OR [mh "Central African Republic"] OR [mh "Chad"] OR [mh "China"] OR [mh "Congo"] OR [mh "Democratic Republic of the Congo"] OR [mh "Democratic Peoples Republic of Korea"] OR [mh "Eswatini"] OR [mh "Ethiopia"] OR [mh "Gabon"] OR [mh "Guinea"] OR [mh "Guinea-Bissau"] OR [mh "Ghana"] OR [mh "India"] OR [mh "Indonesia"] OR [mh "Kenya"] OR [mh "Lesotho"] OR [mh "Liberia"] OR [mh "Malawi "] OR [mh "Mongolia"] OR [mh "Mozambique"] OR [mh "Myanmar"] OR [mh "Namibia"] OR [mh "Nigeria"] OR [mh "Pakistan"] OR [mh "Papua New Guinea"] OR [mh "Philippines"] OR [mh "Russia"] OR [mh "Sierra Leone"] OR [mh "South Africa"] OR [mh "Tanzania"] OR [mh "Thailand"] OR [mh "Uganda"] OR [mh "Vietnam"] OR [mh "Zambia"] OR [mh "Zimbabwe"] OR angola*:ti,ab,kw OR Bangladesh*:ti,ab,kw OR botswan*:ti,ab,kw OR batswan*:ti,ab,kw OR motswan*:ti,ab,kw OR Brazil*:ti,ab,kw OR cambodia*:ti,ab,kw OR cameroon*:ti,ab,kw OR "central africa*":ti,ab,kw OR chad*:ti,ab,kw OR chin*:ti,ab,kw OR congo*:ti,ab,kw OR kinshasa*:ti,ab,kw OR brazzaville*:ti,ab,kw OR "north korea*":ti,ab,kw OR eswatin*:ti,ab,kw OR emaswati*:ti,ab,kw OR swaziland*:ti,ab,kw OR ethiopia*:ti,ab,kw OR gabon*:ti,ab,kw OR ghana*:ti,ab,kw OR guinea*:ti,ab,kw OR india*:ti,ab,kw OR indonesia*:ti,ab,kw OR kenya*:ti,ab,kw OR lesotho*:ti,ab,kw OR sotho:ti,ab,kw OR liberia*:ti,ab,kw OR malawi*:ti,ab,kw OR mongolia*:ti,ab,kw OR mozambi*:ti,ab,kw OR myanmar*:ti,ab,kw OR burm*:ti,ab,kw OR namibia*:ti,ab,kw OR nigeria*:ti,ab,kw OR pakistan*:ti,ab,kw OR "papua new guinea*":ti,ab,kw OR philippine*:ti,ab,kw OR filipino:ti,ab,kw OR russia*:ti,ab,kw OR "sierra leon*":ti,ab,kw OR "south africa*":ti,ab,kw OR tanzania*:ti,ab,kw OR thai*:ti,ab,kw OR uganda*:ti,ab,kw OR vietnam*:ti,ab,kw OR zambia*:ti,ab,kw OR zimbabwe*:ti,ab,kw OR Africa*:ti,ab,kw OR Asia*:ti,ab,kw |
| --- | --- |

**Strings**

1-3 as in the tables above

| 4 | 1 AND 2 AND 3 |
| --- | --- |

| 5 | publication date from Jan 2010 to present |
| --- | --- |

**Web of Science Core Collection**

**P**

| 1 | Tuberculo* OR TB |
| --- | --- |

**I**

| 2 | Diagnos* OR Test* OR Culture* OR Smear* OR Xpert* |
| --- | --- |

**C**

| 3 | angola* OR bangladesh* OR botswan* OR batswan* OR motswan* OR brazil* OR cambodia* OR cameroon* OR "central africa*" OR chad* OR chin* OR congo* OR kinshasa* OR brazzaville* OR "north korea*" OR eswatin* OR emaswati* OR swaziland* OR ethiopia* OR gabon* OR ghana* OR guinea* OR india* OR indonesia* OR kenya* OR lesotho* OR sotho OR liberia* OR malawi* OR mongolia* OR mozambi* OR myanmar* OR burm* OR namibia* OR nigeria* OR pakistan* OR "papua new guinea*" OR philippine* OR filipino OR russia* OR "sierra leon*" OR "south africa*" OR tanzania* OR thai* OR uganda* OR vietnam* OR zambia* OR zimbabwe* OR Africa* OR Asia* |
| --- | --- |

**Clinical Trials**

| 4 | "randomized" OR "randomly" OR "placebo" OR "trial*" |
| --- | --- |

**Strings**

| 1 (title, abstract) | TI=(Tuberculo* OR TB) OR AB=(Tuberculo* OR TB) |
| --- | --- |

| 2 (title, abstract) | TI=(Diagnos* OR Test* OR Culture* OR Smear* OR Xpert*) OR AB=(Diagnos* OR Test* OR Culture* OR Smear* OR Xpert*) |
| --- | --- |

| 3 (title, abstract) | I=(angola* OR bangladesh* OR botswan* OR batswan* OR motswan* OR brazil* OR cambodia* OR cameroon* OR "central africa*" OR chad* OR chin* OR congo* OR kinshasa* OR brazzaville* OR "north korea*" OR eswatin* OR emaswati* OR swaziland* OR ethiopia* OR gabon* OR ghana* OR guinea* OR india* OR indonesia* OR kenya* OR lesotho* OR sotho OR liberia* OR malawi* OR mongolia* OR mozambi* OR myanmar* OR burm* OR namibia* OR nigeria* OR pakistan* OR "papua new guinea*" OR philippine* OR filipino OR russia* OR "sierra leon*" OR "south africa*" OR tanzania* OR thai* OR uganda* OR vietnam* OR zambia* OR zimbabwe* OR Africa* OR Asia*) OR AB=(angola* OR bangladesh* OR botswan* OR batswan* OR motswan* OR brazil* OR cambodia* OR cameroon* OR "central africa*" OR chad* OR chin* OR congo* OR kinshasa* OR brazzaville* OR "north korea*" OR eswatin* OR emaswati* OR swaziland* OR ethiopia* OR gabon* OR ghana* OR guinea* OR india* OR indonesia* OR kenya* OR lesotho* OR sotho OR liberia* OR malawi* OR mongolia* OR mozambi* OR myanmar* OR burm* OR namibia* OR nigeria* OR pakistan* OR "papua new guinea*" OR philippine* OR filipino OR russia* OR "sierra leon*" OR "south africa*" OR tanzania* OR thai* OR uganda* OR vietnam* OR zambia* OR zimbabwe* OR Africa* OR Asia*) |
| --- | --- |

| 4 (title, abstract, author keywords) | TI=("randomized" OR "randomly" OR "placebo" OR "trial*") OR AB=("randomized" OR "randomly" OR "placebo" OR "trial*") OR AK=("randomized" OR "randomly" OR "placebo" OR "trial*") |
| --- | --- |

| 5 | 1 AND 2 AND 3 AND 4 |
| --- | --- |

| 6 | 5 AND Publications from 2010 |
| --- | --- |

### **Table S1: tool for risk of bias evaluation.**

|  | Yes | No | Unclear |
| --- | --- | --- | --- |
| 1. Was the sample frame appropriate to address the target population? (no prespecified population) | □ | □ | □ |
| 1. Were study participants recruited in an appropriate way? (Passive, Consecutive) | □ | □ | □ |
| 1. Did the study avoid inappropriate exclusions for proportion evaluation? | □ | □ | □ |
| 1. Was the sample size adequate? (≥ n=360 TB Pat.) | □ | □ | □ |
| 1. Were the study subjects and the setting described in detail? | □ | □ | □ |
| 1. Was the data analysis conducted with sufficient coverage of the identified sample? | □ | □ | □ |
| 1. Were valid methods used for the identification of TB and applied on all participants? (WHO diagnostic tools & pathways?) | □ | □ | □ |
| 1. Was the term "clinical diagnosis" defined in an appropriate way? | □ | □ | □ |

### **List of variables extracted**

Title, authors, main authors contact details, year of publication, time period of data collection, study design, country/countries of data collection, demographics (age/sex), level of healthcare (primary/secondary), type of patient population (outpatient/inpatient), setting (single site/multi site), eligibility criteria, recruitment (prospective/retrospective), aim of study, population description, diagnostic algorithm, definitions for diagnosis, diagnostic tests used for TB diagnosis, total number of participants screened, total number of participants included, the proportion of clinically diagnosed patients tested by bacteriological means, proportion of patients tested by smear microscopy only, proportion of people living with HIV, proportion of pulmonary TB and extrapulmonary TB, time frame for follow up, description of intervention if applicable, total number of patients with clinical and bacteriological TB diagnoses and in subgroups of HIV status (positive/negative) and TB location (pulmonary/extrapulmonary), data on mortality stratified by TB confirmation status (where provided)

| Study ID | RoB/8* | Des. | Country(-ies) | Start | End | HCL | PTL | Diagnostics available | Population description | SM only in Bact+ | Clinically diagnosed without test | Toatal TB† | Male | Age‡ | HIV | TB location | Follow-up |
| --- | --- | --- | --- | --- | --- | --- | --- | --- | --- | --- | --- | --- | --- | --- | --- | --- | --- |
| Abdullahi 2021 | 7 | CSS | Kenya | 01/2012 | 12/2018 | Mix | Mix | SM, XP, CXR | Adults, started on TB treatment | 48·7% | 30·1% | 12856 | 59·0% | 37 | 29·0% | 12% EPTB | 6M |
| Adewole 2015 | 5 | RCT | Nigeria | - | - | Sec. | Outp. | SM | ≥15y, not on TB treatment, no relevant coexisting medical conditions | 100% | 0% | 150 | 52·7% | 36·5 | 6·7% | PTB only | 2M |
| Åhsberg 2023 | 7 | CRT | Ghana | 10/2019 | 01/2022 | Sec. | Inp. | CU, XP, LAM | Adult PLHIV, symptomatic, severe illness or advanced HIV, mainly urban, not receiving TB treatment in the preceding 60d | 0% | - | 105 | 32·2% | 42 | 100·0% | - |  |
| Atekem 2018 | 6 | CSS | Cameroon | 01/2011 | 12/2012 | Prim. | Outp. | SM |  | 100% | 0% | 895 | 53·5% | 33 | 47·7% | 17·9% EPTB |  |
| Auld 2016 | 7 | CHS | Cambodia | 03/2012 | 06/2013 | Mix | Outp. | SM, CU, XP, CXR | PLHIV, rural, symptomatic | 7·1% | 36·3% | 234 | 46·0% | 37 | 100·0% | PTB only |  |
| Balcha 2014 | 6 | CHS | Ethiopia | 10/2011 | 03/2013 | Prim. | Mix | CU >50%, SM, XP | Adult PLHIV, not on ART, irresp. of TB symptoms, producing sputum | 22·6% | 0% | 158 | 41·0% | 32 | 100·0% | 7·2% EPTB | 6M |
| Beckwith 2021 & Grant 2020 | 6 | RCT | South Africa | 01/2013 | 08/2015 | Sec. | Inp. | SM, CU, XP, CXR | ≥18y, CD4 count ≤150 cells/μL, no relevant coexisting medical conditions, newly diagnosed TB | 11·1% | 100% | 95 | 48·2% | 38·8 | 100·0% | - | 3M |
| Bekele 2018 | 4 | RCT | Ethiopia | 01/2013 | 05/2015 | Mix |  | CU >50%, SM, IGRA | Adults, newly diagnosed PTB, no relevant coexisting medical conditions | 13·8% | 0% | 348 | 57·7% | 30·5 | 0·0% | PTB only |  |
| Bezerra 2020 | 4 | CHS | Brazil | 07/2012 | 06/2019 | Sec. | Inp. | - | Adults, started on TB Treatment | - | - | 148 | 64·9% | 43·3 | 37·2% | 43·3% EPTB | 06/2019 |
| Bjerrum 2015 | 6 | CSS | Ghana | 01/2013 | 03/2014 | Sec. | Mix | SM, CU, XP, CXR, LAM | ≥18 y, CD4 count ≤350 cells/μl, irresp. of TB symptoms, producing sputum, no TB treatment 3M before enrolment | 6% | 0% | 100 | 47·0% | 37 | 100·0% | - | 6M |
| Bock 2018 | 5 | CHS | South Africa | 01/2014 | 11/2015 | Prim. | Outp. | CU, XP, CXR | ≥18y, started ART | 0% | - | 97 | 32·1% | 31 | 100·0% | 16·5% EPTB | 6M |
| Burke 2024 | 7 | RCT | Malawi | 09/2020 | 02/2022 | Sec. | Inp. | CU, XP, CXR LAM, CAD | ≥18y, irresp. of symptoms, no TB treatment 6M before enrolment | - | 0% | 415 | 43·4% | 41 | 100·0% | . | 2M |
| Cattamanchi 2021 | 6 | RCT | Uganda | 10/2018 | 02/2020 | Prim. |  | SM, XP | Adults, PTB, § | 43·5% | - | 824 | 39·9% | 40 | 43·8% | PTB only | 14d |
| Cox 2014 | 7 | RCT | South Africa | 09/2010 | 10/2011 | Prim. | Outp. | SM, CU, XP | Adults, symptomatic, no TB Treatment for >3d before enrolment, § | 16·5% | 2·5% | 506 | 54·7% | - | 58·8% | PTB only | 6M |
| Dave 2013 | 7 | CSS | India | 01/2012 | 09/2012 | Mix | Mix | SM | All TB patients (adults and children) registered | 100% | 0% | 556 | 66·7% | 35 | 4·3% | 13·3% EPTB |  |
| DeCastro 2021 | 4 | RCT | Brazil, Côte d’Ivoire, +3 | 09/2015 | 01/2018 | - |  | SM, CU, XP, LAM, CXR | ≥18y, ART naive, Rifampicin containing TB Treatment initiated max. 8W ago, no relevant coexisting medical conditions | - | - | 457 | 60·2% | 35 | 100·0% | 31·5% EPTB |  |
| Durovni 2014 | 4 | CRT | Brazil | 02/2012 | 10/2012 | Mix | Mix | SM, CU, XP, CXR | Producing sputum, PTB | - | 0% | 4660 | 64·3% | - | 8·9% | PTB only |  |
| Eneogu 2024 | 3 | CSS | Nigeria | 01/2022 | 09/2022 | - | - | - | record in laboratory register, complete data set | - | - | 4823 | - | - | - | - |  |
| Ereso 2024 | 4 | CSS | Ethiopia | 09/2016 | 10/2017 | Mix | - | - | ≥15y, initiated TB treatment | - | - | 755 | 51·2% | - | 3·3% | 29·7% EPTB |  |
| Gebreegziabher 2016 | 6 | CSS | Ethiopia | 10/2013 | 10/2014 | Mix | Mix | SM | ≥15y, newly diagnosed PTB, no TB retreatment cases | 100% | 0% | 706 | 59·9% | 30 | 11·6% | PTB only |  |
| Getahun 2016 | 6 | CSS | Ethiopia | 09/2015 | 11/2015 | - |  | SM | Adults, new TB cases, on DOT at least for 1M | 100% | 0% | 576 | 52·8% | - | - | 38·6% EPTB |  |
| Getiye 2024 | 6 | CSS | Ethiopia | 09/2022 | 10/2022 | Mix | Mix | - | Adults, newly diagnosed PTB attending TB clinics in public health facilities, mainly rural | - | - | 420 | 49·3% | 35·9 | 2·0% | PTB only |  |
| Gupta-Wright 2018 | 7 | RCT | Malawi, South Africa | 10/2015 | 09/2017 | Sec. | Inp. | SM, CU, XP, LAM, CXR | ≥18y, hospitalized, urban and rural, irresp. of  TB symptoms, not on TB treatment for 12M before enrolment | - | - | 474 | 43·2% | 39·6 | 100·0% | - | 2M |
| Hanifa 2016 | 4 | CHS | South Africa | 09/2012 | 03/2014 | - |  | SM, CU, XP, LAM, CXR | ≥18y, CD4 count <200 cells/μl, in HIV care, irresp. of symptoms, no TB treatment within 3M before enrolment | 1·8% | 0% | 56 | 39·1% | 39 | 100·0% | 14·3% EPTB | 3M |
| Hanrahan 2013 | 7 | CHS | South Africa | 07/2011 | 09/2011 | Prim. | Outp. | CU >50%, SM, XP | TB suspects, informal settlement communities | 0% | 0% | 116 | 35·2% | 35 | 69·1% | PTB only | 6M |
| Hemalatha 2023 | 3 | CSS | India | 01/2021 | 12/2021 | Sec. | - | - | PTB and EPTB | - | - | 45 | 55·5% | 53·7 | - | 42·2% EPTB |  |
| Humphrey 2020 | 5 | CHS | Kenya, Uganda , +10 | 01/2012 | 12/2014 | Sec. |  | SM, CU, XP, LAM, CXR | ≥15y, started on TB treatment | 77·9% | 38·5% | 2091 | 56·0% | 36 | 100·0% | 20% EPTB | 12M |
| Jiang 2023 | 5 | CSS | China | 01/2017 | 12/2021 | - | Mix | SM, CU, XP | Not on TB treatment yet, not recorded as “dead”, “treatment failure”, or who were “not evaluated” | - | - | 24265 | 65·5% | 37 | - | - |  |
| Jin 2020 | 5 | TAS | China | 04/2017 | 03/2018 | Sec. | Inp. | CU >50%, XP | Patients with different diseases (infectious and non-infectious) | 0% | 0% | 125 | 72·8% | 45·4 | - | - |  |
| Kaku 2024 | 4 | CSS | Indonesia | 01/2021 | 12/2021 | - | - | XP |  | 0% | 0% | 158 | 57·0% | 48 | 1·2% | PTB only |  |
| Kebede 2021 | 7 | CSS | Ethiopia | 01/2015 | 12/2017 | Sec. | Inp. | SM | ≥15y, started on TB treatment | 100% | 0% | 465 | 54·0% | 35 | 27·4% | 51·6% EPTB |  |
| Kibirige 2023 | 6 | TAS | Uganda | 01/2022 | 01/2023 | Sec. | Outp. | SM | ≥18y, either TB treatment naïve or initiated on TB treatment | 100% | 0% | 232 | 31·0% | 35 | 38·6% | 5% EPTB |  |
| Majella 2021 | 6 | RCT | India | 03/2015 | 06/2016 | Sec. | Mix | SM | New diagnosis of TB, possession of mobile phone | 100% | - | 310 | 69·4% | 39 | 5·8% | 53·9% EPTB |  |
| Manosuthi 2012 | 5 | RCT | Thailand | 10/2009 | 05/2011 | Sec. | Mix | SM, CU, CXR | 18–65y, CD4 count <350 cells/μl, ART naive, no relevant coexisting medical conditions | - | - | 156 | 77·6% | 38 | 100·0% | 53·% EPTB | 6M & 12M |
| McCarthy 2018 & Churchyard 2015 | 5 | CRT | South Africa | 06/2012 | 11/2012 | Prim. | Outp. | SM, CU, XP, CXR | ≥18y, started on TB treatment, negative initial XTEND study sputum results, clinical indication for sputum investigation | 44% | - | 541 | 38·0% | 36 | 62·0% | - | 6M |
| Mishra 2023 | 5 | CSS | India | 04/2019 | 10/2020 | Sec. | Outp. | SM | Adult, on DOTS, new cases of PTB, on treatment for <10 days, no relevant coexisting medical conditions | 100% | 0% | 341 | 76·5% | 30·3 | 0·0% | PTB only |  |
| Mohammed 2020 | 4 | CSS | Ethiopia | 01/2015 | 12/2017 | Mix | Outp. | SM, XP, CXR | TB suspects | - | - | 2483 | - | - | 12·5% | 29% EPTB |  |
| Mupfumi 2014 | 6 | RCT | Zimbabwe | 10/2011 | 06/2012 | Sec. |  | SM, XP, CXR | ≥18y, ART naive initiating ART, not on TB treatment, urban resident, irresp. of TB symptoms | 15·9% | 0% | 88 | 45·3% | 37 | 100·0% | - | 3M |
| Ncube 2019 | 5 | CSS | Zimbabwe | 04/2016 | 10/2017 | Prim. | Outp. | - | ≥15y, newly registered, densely populated poor urban suburb resident | - | - | 1617 | 59·7% | 37 | 67·5% | - |  |
| O'Connor 2017 | 6 | CRT | Lesotho | 04/2013 | 03/2015 | Mix |  | SM, CU, XP, CXR | ≥18y, newly registered for TB treatment | 82·6% | 37·3% | 1233 | 56·7% | 38·6 | 100·0% | 12·8% EPTB |  |
| Padda 2015 | 6 | CSS | India | 01/2011 | 12/2013 | - |  | SM | Put on DOTS | 100% | - | 2571 | - | - | - | 32·3% EPTB |  |
| Peter 2016 & Zijenah 2016 | 6 | RCT | South Africa, Tanzania, +2 | 01/2013 | 10/2014 | Sec. | Inp. | CU >50%, SM, XP, LAM, CXR | ≥18y, symptomatic, severely ill, no TB treatment within 60d before testing | - | 22·5% | 1246 | 48·6% | 37 | 100·0% | - | 2M |
| Prudhivi 2019 | 7 | CHS | India | 01/2014 | 12/2016 | Sec. |  | SM | New and retreatment PTB, data on treatment outcome available | 100% | 0% | 1113 | 66·0% | 47·1 | 23·0% | PTB only | 1Y |
| Qiu 2015 | 6 | RCT | China | 10/2012 | 10/2012 | Sec. |  | CU >50%, SM, CXR, IGRA | TB suspects | - | 12·6% | 597 | 63·5% | 45·2 | 0·0% | 13·4% EPTB |  |
| Rima 2024 | 7 | CSS | Ethiopia | 01/2022 | 08/2022 | Mix | - | SM, XP, CXR | ≥15y, PTB, on DOT, mainly rural, not critically ill | - | - | 393 | 55·0% | - | - | PTB only |  |
| Sachdeva 2015 | 6 | CHS | India | 03/2012 | 12/2013 | Prim. |  | SM, XP, CXR | Symptomatic, producing sputum | 8·7% | - | 17587 | 64·7% | 39·9 | - | PTB only |  |
| Seid 2018 | 5 | CSS | Ethiopia | 04/2016 | 01/2017 | Mix | Mix | SM, XP, CXR | ≥18y, newly diagnosed TB, <15d of TB treatment, not critically ill | 87·2% | 0% | 382 | 52·6% | 37·1 | 20·4% | 40·3% EPTB |  |
| Shivalingaiah 2024 | 5 | CHS | India | 01/2022 | 12/2022 | - | - | - | All TB cases ≥18y, registered for treatment at study site | - | - | 516 | 54·6% | 35·9 | 1·7% | 42·8% EPTB |  |
| Shrivastava 2013 | 6 | CHS | India | 08/2010 | 01/2011 | Sec. |  | SM | Symptomatic | 100% | 0% | 61 | 57·4% |  | 10·2% | 12·5% EPTB |  |
| Songkhla 2019 | 6 | CHS | Thailand | 12/2015 | 03/2017 | Sec. | Mix | CU >50%, SM, LAM, CXR | Adults, CD4 cell count ≤200/μL, symptomatic, irresp. of sputum producability, no TB Treatment within 3M before enrolment | - | 0% | 137 | 62·5% | 39 | 100·0% | - |  |
| Theron 2014 | 8 | RCT | South Africa, Zimbabwe, +2 | 04/2011 | 03/2012 | Mix | Outp. | CU >50%, SM, XP, CXR | ≥18y, periurban, symptomatic, no treatment in previous 60d, producing sputum | 3·3% | 0% | 645 | 57·2% | 37 | 59·6% | PTB only | 6M |
| Weber 2018 | 6 | CHS | India | 01/2016 | 07/2016 | Sec. | Mix | SM, CU, CXR | ≥ 16y, presumptive TB | - | - | 285 | 77·5% | 40 | 14·0% | 34·7% EPTB |  |
| Zerihun 2023 | 7 | CSS | Ethiopia | 01/2017 | 12/2019 | Prim. | Outp. | SM | ≥18y, PTB, newly started on TB treatment, mainly urban | 100% | 0% | 636 | 56·8% | 37·5 | 16·7% | PTB only |  |

### **Table S2: Extended baseline table of included studies**

Des.: Study design, HCL: healthcare level, PTL: patientlevel, SM: smear microscopy, Bact+: bacteriologocally positively tested, CSS: cross sectional, RCT: randomised controlled trial, DAS: diagnostic accuracy study, CRT: cluster randomized trial, Prim.: primary, Sec.: secondary, Mix: mixed, Outp.: outpatients, Inp.: inpatients, CU: culture, XP: X-pert, CXR: chest X-ray, IGRA: interferon gamma release assay, LAM: lipoarabinomannan on urine, CAD: digital chest X-ray with computer-aided diagnosis (dCXR-CAD, CAD4TBv6, Delft, Netherlands), PLHIV: people living with HIV, ART: antiretroviral therapy, PTB: pulmonary TB, EPTB: extrapulmonary TB, DOTS: direct observed therapy, short course, DOT: directly observed therapy, *Risk of bias score out of 8, 8 indicating lowest risk of bias , †total amount of patients diagnosed with TB, ‡ age reported in mean or median according to reporting in study, § we included people set on treatment only

**Table S3: Risk of bias assessment results**

| Study ID | 1.Popula-tion | 2. Recruit-ment | 3. Exclusion reasons | 4. Sample size | 5. Descrip-tion | 6. Coverage | 7. Diagnostic method | 8.Defini-tion | Score out of 8 | Final Decision on Bias |
| --- | --- | --- | --- | --- | --- | --- | --- | --- | --- | --- |
| Abdullahi 2021 | Yes | Yes | Yes | Yes | Yes | Yes | No | Yes | 7 | Low |
| Adewole 2015 | Yes | Yes | No | No | Yes | Yes | No | Yes | 5 | High |
| Åhsberg 2023 | Yes | Yes | Yes | No | Yes | Yes | Yes | Yes | 7 | Low |
| Atekem 2018 | Yes | Yes | Yes | Yes | No | Yes | No | Yes | 6 | High |
| Auld 2016 | Yes | Yes | Yes | No | Yes | Yes | Yes | Yes | 7 | Low |
| Balcha 2014 | Yes | Yes | Yes | No | Yes | Yes | No | Yes | 6 | High |
| Beckwith 2021 & Grant 2020 | Yes | Yes | No | No | Yes | Yes | Yes | Yes | 6 | High |
| Bekele 2018 | Yes | Unclear | No | No | No | Yes | Yes | Yes | 4 | Very High |
| Bezerra 2020 | Yes | Yes | No | No | Yes | Yes | Unclear | Unclear | 4 | Very High |
| Bjerrum 2015 | Yes | Yes | Yes | No | Yes | Yes | Yes | No | 6 | High |
| Bock 2018 | Yes | Yes | Yes | No | Yes | Yes | Unclear | No | 5 | High |
| Burke 2024 | Yes | Yes | Yes | No | Yes | No | Yes | Yes | 6 | High |
| Cattamanchi 2021 | Yes | Yes | Yes | Yes | Yes | Yes | No | Unclear | 6 | High |
| Cox 2014 | Yes | Yes | Yes | Yes | Yes | Yes | Yes | Unclear | 7 | Low |
| Dave 2013 | Yes | Yes | Yes | Yes | Yes | Yes | No | Yes | 7 | Low |
| DeCastro 2021 | No | Unclear | No | Yes | No | Yes | Yes | Yes | 4 | Very High |
| Eneogu 2024 | Unclear | Unclear | Yes | Yes | No | Yes | Unclear | Unclear | 3 | Very High |
| Ereso 2024 | Yes | Yes | No | Yes | No | Yes | Unclear | Unclear | 4 | High |
| Durovni 2014 | Yes | Yes | Yes | Yes | No | No | Unclear | Unclear | 4 | High |
| Gebreegziabher 2016 | Yes | Yes | No | Yes | Yes | Yes | No | Yes | 6 | HIgh |
| Getahun 2016 | Yes | Yes | Yes | Yes | No | Yes | No | Yes | 6 | High |
| Getiye 2024 | Yes | Yes | Yes | Yes | Yes | Yes | Unclear | Unclear | 6 | High |
| Gupta-Wright 2018 | Yes | Yes | Yes | Yes | Yes | Yes | No | Yes | 7 | Low |
| Hanifa 2016 | Yes | Unclear | No | No | No | Yes | Yes | Yes | 4 | High |
| HanrahanColleen 2013 | Yes | Yes | Yes | No | Yes | Yes | Yes | Yes | 7 | Low |
| Hemalatha 2023 | Yes | Yes | Unclear | No | No | Yes | Unclear | Unclear | 3 | Very High |
| Humphrey 2020 | No | Yes | Yes | Yes | Yes | Yes | No | No | 5 | High |
| Jiang 2023 | Yes | Yes | No | Yes | No | Yes | Yes | Unclear | 5 | High |
| Jin 2020 | Yes | Unclear | Unclear | No | Yes | Yes | Yes | Yes | 5 | High |
| Kaku 2024 | Yes | Yes | Unclear | No | No | Unclear | Yes | Yes | 4 | Very High |
| Kebede 2021 | Yes | Yes | Yes | Yes | Yes | Yes | No | Yes | 7 | Low |
| Kibirige 2023 | Yes | Yes | Yes | No | Yes | Yes | No | Yes | 6 | High |
| Majella 2021 | Yes | Yes | Yes | No | Yes | Yes | No | Yes | 6 | High |
| Manosuthi 2012 | Yes | Unclear | No | No | Yes | Yes | Yes | Yes | 5 | High |
| McCarthy 2018 & Churchyard 2015 | Yes | No | Yes | Yes | Yes | No | No | Yes | 5 | High |
| Mishra 2023 | Yes | Yes | No | No | Yes | Yes | No | Yes | 5 | High |
| Mohammed 2020 | Yes | Yes | Yes | Yes | No | No | Unclear | Unclear | 4 | High |
| Mupfumi 2014 | Yes | Yes | Yes | No | Unclear | Yes | Yes | Yes | 6 | High |
| Ncube 2019 | Yes | Yes | Yes | Yes | No | Yes | Unclear | Unclear | 5 | High |
| O'Connor 2017 | Yes | Yes | Yes | Yes | Yes | No | No | Yes | 6 | High |
| Padda 2015 | Yes | Yes | Yes | Yes | No | Yes | No | Yes | 6 | High |
| Peter 2016 & Zijenah 2016 | Yes | No | No | Yes | Yes | Yes | Yes | Yes | 6 | High |
| Prudhivi 2019 | Yes | Yes | Yes | Yes | Yes | Yes | No | Yes | 7 | Low |
| Qiu 2015 | Yes | Yes | Unclear | Yes | No | Yes | Yes | Yes | 6 | High |
| Rima 2024 | Yes | Yes | Yes | Yes | No | Yes | Yes | Yes | 7 | Low |
| Sachdeva 2015 | Yes | Yes | Yes | Yes | No | Yes | Yes | No | 6 | High |
| Seid 2018 | Yes | Yes | No | Yes | Yes | Yes | No | No | 5 | High |
| Shivalingaiah 2024 | Yes | Yes | Yes | Yes | No | Yes | Unclear | Unclear | 5 | High |
| Shrivastava 2013 | Yes | Yes | Yes | No | Yes | Yes | No | Yes | 6 | High |
| Songkhla 2019 | Yes | Yes | Yes | No | Yes | Yes | Yes | No | 6 | High |
| Theron 2014 | Yes | Yes | Yes | Yes | Yes | Yes | Yes | Yes | 8 | Low |
| Weber 2018 | Yes | Yes | Yes | No | Yes | Yes | Unclear | Yes | 6 | High |
| Zerihun 2023 | Yes | Yes | Yes | Yes | Yes | Yes | No | Yes | 7 | Low |

We assessed risk of bias using a bespoke tool based on the JBI risk of bias tool for prevalence studies and diagnostic accuracy studies 1: Was the sample frame appropriate to address the target population?, 2: Were study participants recruited in an appropriate (passive & consecutive) way?, 3: Did the study avoid inappropriate exclusions for proportion evaluation?, 4: Was the sample size adequate? (n≥360), 5: Were the study subjects and the setting described in detail?, 6: Was the data analysis conducted with sufficient coverage of the identified sample?, 7: Were valid methods used for the identification of TB and applied on all participants?, 8: Was the term "clinical diagnosis" defined in an appropriate way?

###

### **Figure S1: Meta-analysis of proportion of clinical TB diagnosis for all studies**

Inverse variance random effects meta-analysis on the proportion of clinically diagnosed TB patients in all patients diagnosed as TB with data on confirmation status available. Studies sorted by proportion. On the left side: Risk of bias assessment score out of 8, absolute number of TB patients and absolute number of clinically diagnosed TB Patients. On the right: the proportion of clinically diagnosed patients per individual study with 95% CIs and weight within meta-analysis. On the bottom left: estimates for heterogeneity. On the bottom right: pooled summary estimate for proportion with 95% CI. Where clinical trial arms have been presented separately, the description of the arm is presented after the citation. ROB risk of bias, SOC Standard of Care, Smear is sputum smear microscopy, Xpert is Xpert MTB/RIF or Xpert MTB/RIF Ultra arm, LAM is Urine lipoarabinaomannan (LAM) testing, CAD is digital chest X-ray with computer-aided diagnosis and Truenat a real time PCR device.

###

### **Figure S2: Meta-analysis of proportion of clinical TB diagnosis in extrapulmonary TB**

Inverse variance random effects meta-analysis on the proportion of clinically diagnosed TB patients among patients with extrapulmonary TB. On the left: risk of bias assessment score out of 8, absolute numbers of EPTB patients and clinically diagnosed EPTB patients. On the right the proportion, 95% CIs and weight. Summary measures and heterogeneity analysis below.

###

###

### **Figure S3: Meta-analysis of proportion of clinical TB diagnosis in pulmonary TB**

Inverse variance random effects meta-analysis on the proportion of clinically diagnosed TB patients among pulmonary TB patients. On the left: risk of bias assessment score out of 8, absolute numbers of PTB patients and clinically diagnosed PTB patients. On the right the proportion, 95% CIs and weight. Summary measures and heterogeneity analysis below. ROB risk of bias, PTB Pulmonary TB, EPTB Extrapulmonary TB, SOC Standard of Care, Smear is sputum smear microscopy, Xpert is Xpert MTB/RIF or Xpert MTB/RIF Ultra arm

###

### **Figure S4: Meta-analysis of proportion of clinical TB diagnosis in people living with HIV**

Inverse variance random effects meta-analysis on the proportion of clinically diagnosed TB patients among people living with HIV (PLHIV). On the left: risk of bias assessment score out of 8, absolute numbers of PLHIV and clinically diagnosed PLHIV. On the right the proportion, 95% CIs and weight. Summary measures and heterogeneity analysis below. PTB Pulmonary TB, EPTB Extrapulmonary TB, SOC Standard of Care, Smear is sputum smear microscopy, Xpert is Xpert MTB/RIF or Xpert MTB/RIF Ultra arm, LAM is Urine lipoarabinaomannan (LAM) testing, CAD is digital chest X-ray with computer-aided diagnosis.

###

###

### **Figure S5: Meta-analysis of proportion of clinical TB diagnosis in people not living with HIV**

Inverse variance random effects meta-analysis on the proportion of clinically diagnosed TB patients among patients tested negative for TB. On the left: risk of bias assessment score out of 8, absolute numbers of HIV negative patients and clinically diagnosed HIV negative patients. On the right the proportion, 95% CIs and weight. Summary measures and heterogeneity analysis below. ROB risk of bias, Smear is sputum smear microscopy, Xpert is Xpert MTB/RIF or Xpert MTB/RIF Ultra arm.

###

###

### **Figure S6: Meta-analysis of proportion of clinical TB diagnosis by healthcare level**

Inverse variance random effects meta-analysis on the proportion of clinically diagnosed TB patients in subgroups of primary and secondary health care. Test for subgroup differences (random effects model): between groups: p-value: 0·0116. On the left absolute number of clinically diagnosed TB and all TB patients with data on confirmation status available. On the right the proportion, 95% CIs and weight. Summary measures, heterogeneity analysis and testing between subgroups below. ROB risk of bias, SOC Standard of Care, Smear is sputum smear microscopy, Xpert is Xpert MTB/RIF or Xpert MTB/RIF Ultra arm, LAM is Urine lipoarabinaomannan (LAM) testing, CAD is digital chest X-ray with computer-aided diagnosis.

###

### **Figure S7: Meta-analysis of proportion of clinical TB diagnosis in subgroups by clinical setting**

Inverse variance random effects meta-analysis on the proportion of clinically diagnosed TB patients in subgroups of inpatients and outpatients. Test for subgroup differences (random effects model): between groups: p-value: 0·0232. On the left absolute number of clinically diagnosed TB and all TB patients with data on confirmation status available. On the right the proportion, 95% CIs and weight. Summary measures, heterogeneity analysis and testing between subgroups below. SOC Standard of Care, Smear is sputum smear microscopy, Xpert is Xpert MTB/RIF or Xpert MTB/RIF Ultra arm, LAM is Urine lipoarabinaomannan (LAM) testing, CAD is digital chest X-ray with computer-aided diagnosis.

###

### **Figure S8: Meta-analysis of proportion of clinical TB diagnosis by availability of bacteriologial testing**

Inverse variance random effects meta-analysis on the proportion of clinically diagnosed TB patients in subgroups of studies not testing everybody bacteriologically and those testing everybody bacteriologically. Test for subgroup differences (random effects model): between groups: p-value: < 0·0001. On the left absolute number of clinically diagnosed TB and all TB patients with data on confirmation status available. On the right the proportion, 95% CIs and weight. Summary measures, heterogeneity analysis and testing between subgroups below.

### **Figure S9: Meta-analysis of proportion of clinical TB diagnosis by diagnostic method available**

Inverse variance random effects meta-analysis on the proportion of clinically diagnosed TB patients in subgroups of studies using smear only and those using Xpert MTB/RIF (Xpert, Cepheid, USA) only. Test for subgroup differences (random effects model): between groups: p-value: 0·6162. The Xpert in study name, labels the study as intervention arm of a controlled trial with diagnostic intervention to reduce the proportion of clinical TB. There was only one study using Xpert only without being part of a clinical trial. On the left absolute number of clinically diagnosed TB and all TB patients with data on confirmation status available. On the right the proportion, 95% CIs and weight. Summary measures, heterogeneity analysis and testing between subgroups below.

###

### **Figure S10: Meta-analysis of proportion of clinical TB diagnosis by risk of bias assessment**

Inverse variance random effects meta-analysis on the proportion of clinically diagnosed TB patients in subgroups of risk of bias categories. Score 7-8: low risk, score 5-6: high risk, score ≤4: very high risk. Test for subgroup differences (random effects model): between groups: p-value: 0·8336. On the left absolute number of clinically diagnosed TB and all TB patients with data on confirmation status available. On the right the proportion, 95% CIs and weight. Summary measures, heterogeneity analysis and testing between subgroups below. SOC Standard of Care, Smear is sputum smear microscopy, Xpert is Xpert MTB/RIF or Xpert MTB/RIF Ultra arm, LAM is Urine lipoarabinaomannan (LAM) testing, CAD is digital chest X-ray with computer-aided diagnosis and Truenat a real time PCR device.

###

### **Figure S11: Meta-regression of proportion of clinical TB diagnosis over mean year of study period**

y-axis is logit transformed proportion of clinical TB within all TB, x-axis is the mean year of the study period, Mixed effects model, n=57 studies, p = 0·5051, *I^2^*= 99·22%. Coefficient -0.02 (95% CI -0.08-0.05).

###

### **Figure S12: Funnel Plot on identified studies**

All n=63 studies resp. study arms in proportion analysis included.

###

### **Figure S13: Meta-analysis of mortality risk by risk of bias assessment**

Random effects inverse variance meta-analysis on mortality risk ratio (event = death of a patient) of clinically diagnosed TB patients compared to bacteriologically confirmed TB patients with data on confirmation status available. Analyzed in subgroups of risk of bias categories. Studies sorted by RR. Absolute numbers on the left. RR per study with 95% CIs and weight on the right. Pooled RR with 95% CI displayed below as well as heterogeneity assessment.

###

### **Figure S14: Meta-regression of mortality risk over time**

Meta-regression on RR of mortality (y-axis) over mean year of the study period (x-axis). Mixed effects model, n=10 studies, p=0·0039, I^2=66·13%. Coefficient 0.21 (95% CI 0.09-0.34).

### **Table S4: Diagnostics used in study arms in clinical trials with an intervention aiming at the reduction of the proportion of clinical TB**

| Study ID | Baseline arm diagnostics | Intervention arm diagnostics | Intervention description | Country (-ies) | HIV status | other information |
| --- | --- | --- | --- | --- | --- | --- |
| Burke 2024 SOC vs. CAD | Culture, Xpert MTB/RIF, CXR, Alere Determine TB-LAM, in routine care setting | Culture, Xpert MTB/RIF, CXR, Alere Determine TB-LAM, dCXR-CAD, SILVAMP-LAM | Adding computer aided CXR and SILVAMP LAM mandatorily as enhanced diagnostics | Malawi | 100% HIV positive | Decision makers unblinded |
| Cox 2014 SOC vs. Xpert | smear microscopy (95·5%), culture at indication (45·8%), Xpert (6%) | Xpert MTB/RIF (85,4%), culture at indication (48·3%), smear (68·8%) | replacing smear by Xpert as initial test in diagnostic algorithm | South Africa | 58·8% HIV positive | Clinic staff and investigators unblinded |
| Durovni 2014 Smear vs. Xpert | smear microscopy, culture? CXR? | Xpert MTB/RIF, culture? CXR? | replacing smear by Xpert as initial test in diagnostic algorithm | Brazil | 8·9%HIV positive | physicians unblinded |
| Eneogu 2024 SOC vs Truenat | no information | Adding Truenat real time PCR device to routine care | multicentre study adding Truenat PCR to assigned facilities | Nigeria | no information |  |
| Gupta-Wright 2018 SOC vs. LAM | Xpert MTB/RIF (56%), smear microscopy (14%), culture (10%), CXR(48%), no urine testing | Xpert MTB/RIF (56%), smear microscopy (16%), culture (11%), CXR(48%), Alere Determine TB-LAM Ag assay on urine (99%), Urine Xpert (99%) | adding urine LAM & urine Xpert | Malawi, South Africa | 100% HIV positive | double blinded |
| Mupfumi 2014 Smear vs. Xpert | fluorescece microscopy, CXR | Xpert MTB/RIF, CXR | replacing smear by Xpert | Zimbabwe | 100% HIV positive | physicians unblinded |
| Peter 2016 SOC vs. LAM | Xpert MTB/RIF (44%), culture (90%), CXR (95%), Sonography/CT-scan(35%), smear microscopy | Xpert MTB/RIF (46%), culture (93%), CXR (95%), Sonography/CT-scan (36%), smear microscopy, Alere Determine urine LAM | adding urine LAM | South Africa, Tanzania, Zambia, Zimbabwe | 100% HIV positive | physicians unblinded |
| Sachdeva 2015 Smear vs. Xpert | smear microscopy, culture, line probe assay | Xpert MTB/RIF, smear microscopy, culture, line probe assay | adding Xpert to smear | India | no information |  |
| Theron 2014 Smear vs. Xpert | culture (100%), smear microscopy (99%), CXR (100%), Alere Determine TB-LAM Ag on urine | culture (100%), Xpert MTB/RIF (99%), CXR (100%), Alere Determine TB-LAM Ag on urine | replacing smear by Xpert | South Africa, Zimbabwe, Zambia, Tanzania | 59·6% HIV positive | physicians unblinded |
| Åhsberg 2023 SOC vs. LAM | Xpert MTB/RIF (62%), CXR (63·7%) | Xpert MTB/RIF (73%), CXR (81%), Alere Determine TB-LAM Ag on urine | adding urine LAM | Ghana | 100% HIV positive |  |

SOC: standard of care, Xpert: Xpert MTB/RIF, CXR: chest x-ray, LAM: lipoarabinomannan on urine, dCXR-CAD: digital chest x-ray computer aided diagnosis (CAD4TB v6 software, Delft imagining, Netherlands), Truenat: Truenat real time PCR device (Molbio Diagnostics, Verna, India)
